# Cancer-derived exosomes: mediators of immune crosstalk and emerging targets for immunotherapy

**DOI:** 10.3389/fimmu.2025.1679934

**Published:** 2025-10-09

**Authors:** Ridwan Mahamed, Bernice Monchusi, Clement Penny, Sheefa Mirza

**Affiliations:** ^1^ Department of Internal Medicine, Faculty of Health Sciences, University of the Witwatersrand, Johannesburg, South Africa; ^2^ Synthetic Nanobiotechnology and Biomachines, Synthetic Biology and Precision Medicine Centre, Future Production Chemicals Cluster, Council for Scientific and Industrial Research, Pretoria, South Africa

**Keywords:** cancer-derived exosomes, immune-crosstalk, immune-modulation, immunotherapy, tumor microenvironment

## Abstract

Exosomes, nanoscale extracellular vesicles secreted by various cell types, play pivotal roles in intercellular communication. In cancer, tumor-derived exosomes—referred to as cancer-derived exosomes (CDEs)—have emerged as critical regulators of immune evasion, tumor progression, and therapy resistance within the tumor microenvironment (TME). CDEs modulate immune cell function through the transfer of immunosuppressive proteins, cytokines, and non-coding RNAs, ultimately reprogramming immune surveillance mechanisms. This review provides an in-depth analysis of how CDEs influence major immune cell subsets—including T cells, B cells, NK cells, dendritic cells, macrophages, and myeloid-derived suppressor cells—thereby establishing an immunosuppressive TME. We also explore the potential of immune cell-derived exosomes (IDEs) as emerging immunotherapeutic tools capable of counteracting the suppressive effects of CDEs. Furthermore, we highlight exosome engineering strategies aimed at improving therapeutic cargo delivery, tumor targeting, and antitumor immune activation. Finally, we discuss how exosome profiling offers promise in liquid biopsy diagnostics and how integration with 3D tumor models and advanced bioengineering can accelerate the clinical translation of exosome-based cancer immunotherapies.

## Introduction

Exosomes, a subtype of extracellular vesicles ranging between 30 and 100 nm, play a crucial role in cell-to-cell communication by transporting proteins, lipids, and nucleic acids reflective of the state of the originating cell (1; 2, 3). Among their various physiological functions, cancer cell exosomes referred to as cancer-derived exosomes (CDEs) have attracted growing interest for their involvement in tumor progression, immune evasion, and metastasis (4, 5). These vesicles interact intricately with immune cells, promoting immunosuppression in the tumor microenvironment and contributing to cancer hallmarks such as immune escape, largely through mechanisms such as exosomal PD-L1-mediated T cell inhibition (6).

Beyond their physiological role, exosomes have gained attention due to their clinical potential in cancer diagnostics, prognosis, and therapeutic monitoring. Their stability in bodily fluids and ability to carry tumor-specific biomarkers make them suitable candidates for liquid biopsies. Biomolecules such as exosomal PD-L1 and miRNAs have shown utility in predicting response to immune checkpoint inhibitors and tracking disease progression in cancers such as melanoma, breast, ovarian, and bladder cancer (7–10).

Recent findings also reveal that cancer therapies such as chemotherapy and radiation therapy can significantly alter the molecular composition and release of tumor-derived exosomes. These post-therapeutic changes can enhance tumor aggressiveness or signal treatment efficacy, depending on the context (11, 12). For example, chemotherapeutics such as paclitaxel and melphalan have been shown to increase exosome release *in vitro* (12, 13), while clinical samples from patients with leukemia and head and neck cancer show reduced exosomal proteins after treatment (14, 15). These discrepancies highlight the complex and context-dependent nature of exosome biology in the response to treatment.

To leverage the full therapeutic potential of exosomes, researchers are engineering immune and tumor-derived exosomes to deliver therapeutic agents such as siRNAs, chemotherapeutic drugs, and immune agonists (16). Various loading techniques, including electroporation, sonication, and surface conjugation, have improved cargo specificity and delivery efficiency (17). Engineered exosomes have been shown to cross biological barriers and target tumor sites with minimal toxicity (18, 19), but their clinical translation still faces hurdles such as standardization, targeting specificity, and large-scale production. This review explores the immunomodulatory functions of CDEs, their potential as biomarkers, and the engineering strategies aimed at overcoming current therapeutic limitations. To further assess the functional relevance and therapeutic impact of engineered exosomes, advanced 3D tumor models, such as spheroids, are emerging as valuable tools that more accurately recapitulate the tumor microenvironment compared to traditional 2D cultures.

## Cancer hallmarks and tumor microenvironment

**Figure 1:**
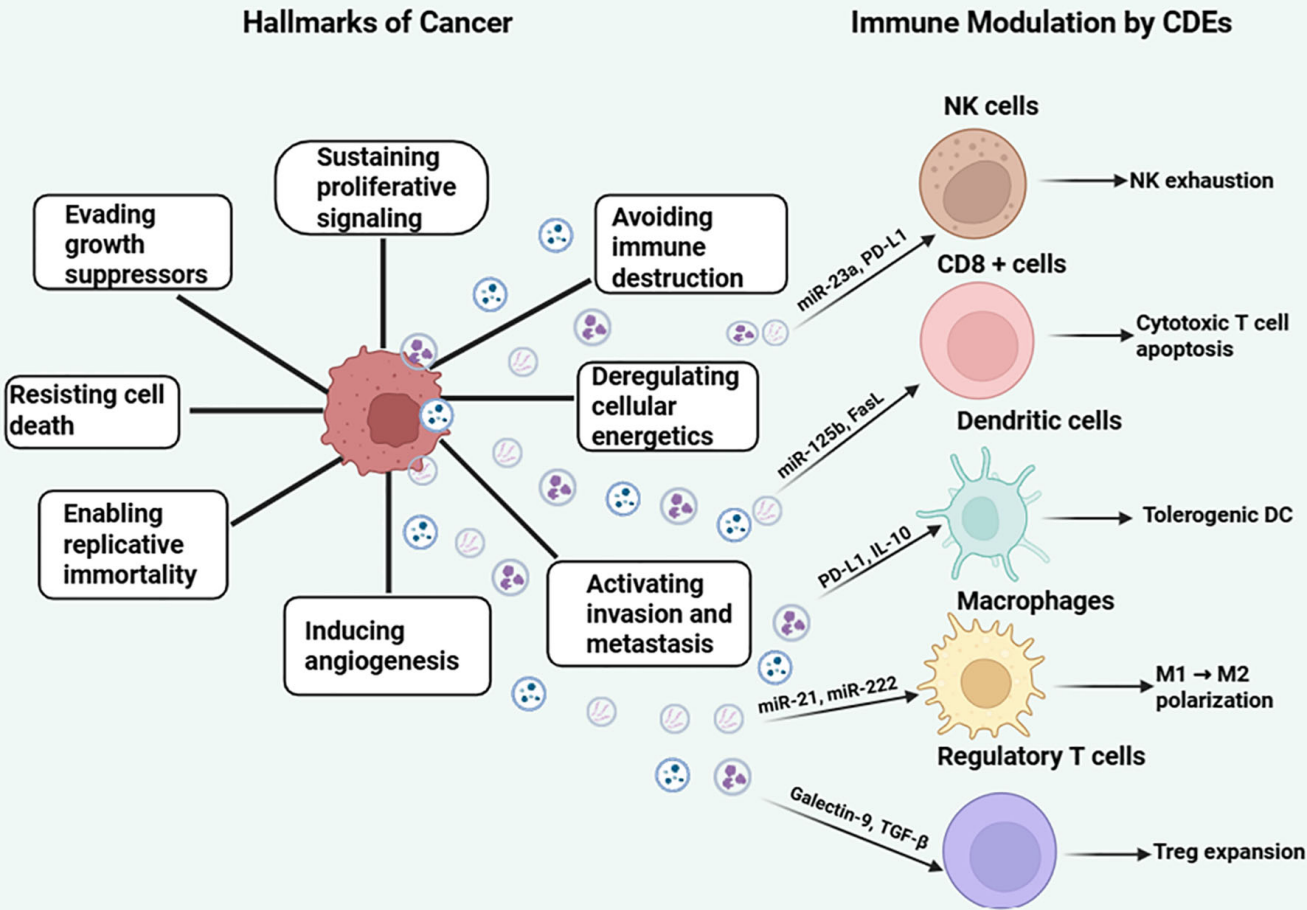
Hallmarks of cancer and immunomodulatory roles of cancer-derived exosomes (CDEs). The central cancer cell is surrounded by the eight classical hallmarks of cancer, including sustained proliferative signaling, evasion of growth suppressors, resistance to cell death, replicative immortality, induction of angiogenesis, activation of invasion and metastasis, deregulated cellular energetics, and avoidance of immune destruction. Cancer-derived exosomes (CDEs) are shown as vesicles released from the cancer cell, carrying immunosuppressive cargo such as miRNAs (e.g., miR-23a, miR-125b), proteins (PD-L1, Galectin-9, FasL), and cytokines (TGF-β, IL-10). These exosomes interact with key immune cells—natural killer (NK) cells, CD8^+^ T cells, dendritic cells (DCs), macrophages, and regulatory T cells (Tregs)—to induce NK exhaustion, cytotoxic T cell apoptosis, Treg expansion, M1-to-M2 macrophage polarization, and tolerogenic DC phenotypes. The left panel illustrates the intrinsic hallmarks of cancer, while the right panel emphasizes the immunomodulatory effects of exosomal signaling on immune evasion, highlighting exosomes as mediators of tumor progression. Figure was designed using BioRender.com.

Cancer cells exploit intercellular communication similarly to healthy cells, but they use it to promote their growth by inhibiting cells that oppose them or activating regulators of cancer hallmarks. These hallmarks include immune evasion, sustained proliferation, metastasis, replicative immortality, angiogenesis, and apoptosis avoidance (20, 21). To survive harsh environments, cancer cells adopt “enabling characteristics” that maintain malignancy and create favorable conditions for tumor progression and metastasis (21). Within the tumor microenvironment (TME), cancer cells continuously maintain these hallmarks by releasing cancer-derived exosomes (CDEs), which regulate surrounding cells and adapt to the hostile TME. Therapeutic strategies can target CDE cargo production to disrupt hallmark maintenance or enhance immune cell function to counteract these cancer-promoting signals (**Figure 1**).

TME is a central hub where cancer hallmarks are enabled, providing favorable conditions for cancer cells while being hostile to normal host cells (21). It comprises cancer-associated fibroblasts (CAFs), immune and stromal cells, blood vessels, and extracellular vesicles (EVs), all of which coordinate to support metastasis and immune evasion through exosome-mediated signaling (21). Exosomes also facilitate tumor innervation via axonogenesis.

Tumor cells reprogram their metabolism toward glycolysis to fuel proliferation by upregulating the output of glucose transporters, and this promotes lactate production leading to the release of protons that acidify the TME and enhance exosomal cargo exchange (22). These exosomes carry factors like DLL4, TGF-β, and Tspan8 that promote angiogenesis and tumor progression (22). Additionally, fibroblasts are reprogrammed into CAFs, further supporting metastasis. Targeting the acidic conditions of the TME by navigating through anti-TME strategies aimed at increasing the pH may provide a therapeutic strategy by altering exosomal cargo profiles (22).

## The building blocks of exosomes

Initially, EVs were described as fragments released by cells ubiquitously; however, it was only until the 1980s that exosomes were characterized as ‘cellular waste units’ which govern communication between cells (23). Subsequently, exosomes were stumbled upon in a study in 1983 where transferrin receptors (TfRs) migrated from the plasma membrane to mature reticulocytes, where they eventually reassembled into small vesicles within these cells (24). The discovery of exosomes marked a turning point in molecular biology as they revolutionized the previously held stance that they were solely for removing cellular garbage, to being the pioneers of cell-cell communication (25). In the past 20 years, exosomes have been progressively characterized and are gaining attention in therapeutics; however, as much as they have potential in therapeutics, their signaling nature is likened to that of a double-edged sword, as they also play a pathological role in diseases like cancer. Thus, understanding the physiological and pathological fate of exosomes requires a detailed exploration of their biogenesis.

**Figure 2:**
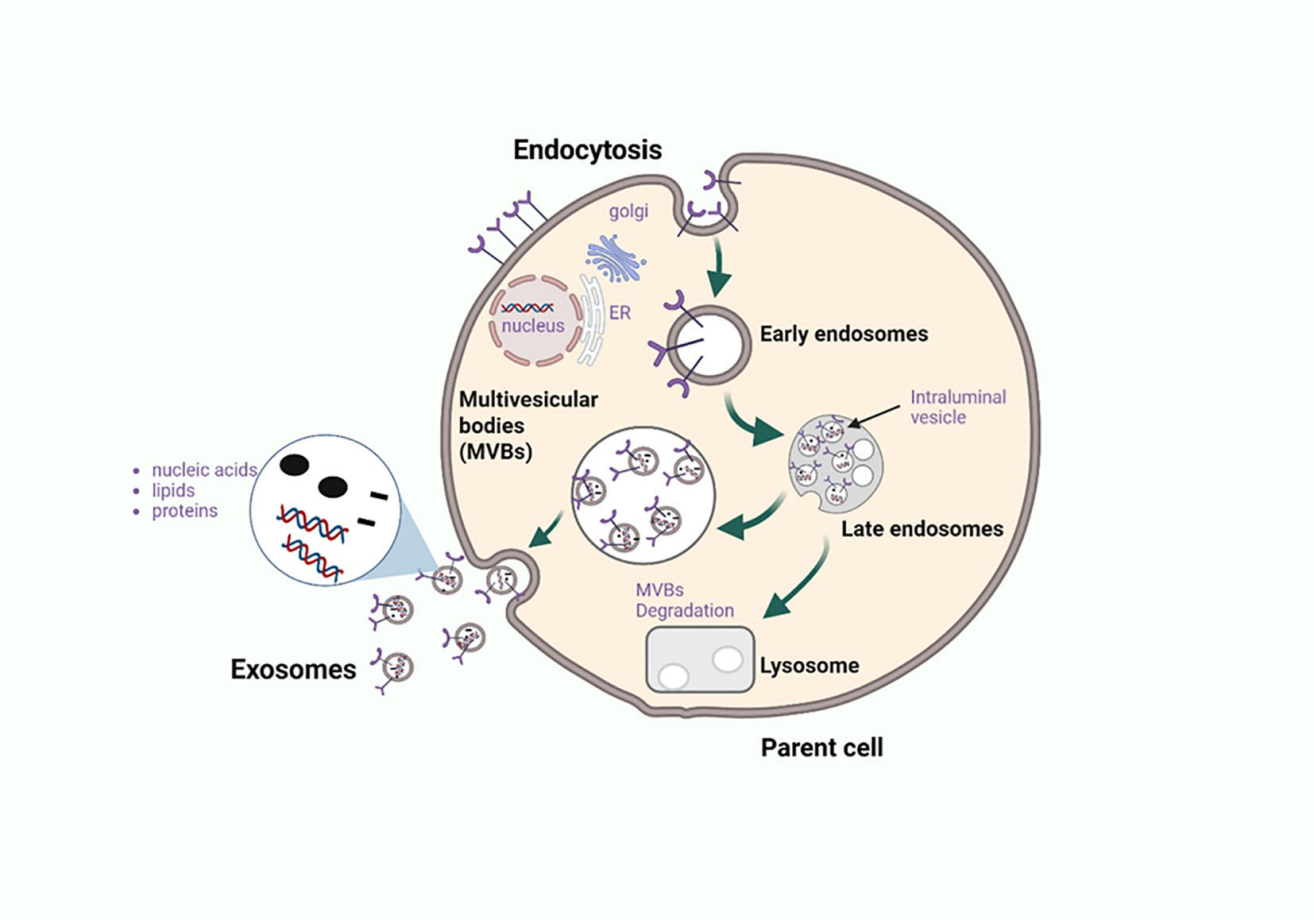
Biogenesis and exosome release from the parent cell. Exosomes are nanoscale extracellular vesicles formed through the endosomal trafficking pathway, beginning with the invagination of the plasma membrane to generate early endosomes. These early endosomes internalize diverse biomolecules such as proteins, nucleic acids, and lipids which are further sorted during maturation into multivesicular bodies (MVBs) or late endosomes. Within MVBs, inward budding of the limiting membrane generates intraluminal vesicles (ILVs) that are selectively loaded with cargo. MVBs can fuse with lysosomes for degradation, particularly when carrying damaged or incomplete cellular components, or merge with the plasma membrane to release ILVs as exosomes into the extracellular space. In the context of cancer, exosomes enriched with immunomodulatory proteins and nucleic acids act as critical mediators of immune crosstalk, promoting tumor progression, immune evasion, and systemic signaling. Elucidating the mechanisms of cargo sorting and release provides insight into novel therapeutic targets aimed at modulating exosome content or blocking their immunosuppressive functions. Figure was designed using BioRender.com.

Exosome biogenesis is triggered when cell cargo undergoes endocytosis within a cell, and the vesicle that buds into the plasma membrane is known as the early endosome (**Figure 2**) (26). At this stage, primary sorting takes place via the endosomal sorting complexes required for transport (ESCRT) and the fate of the cargo to be delivered is determined (26, 27). The main pathway of exosome biogenesis is the classic pathway that uses ESCRT complexes to release exosomes (27). ESCRTs are a group of proteins that localize on the membrane of multivesicular bodies (MVBs) to organize cargo and release intraluminal vesicles (ILVs), which later form exosomes carrying cargo to their designated target cells (27). There are four different networks within ESCRT which are ESCRT-0, ESCRT-I, ESCRT-II, and ESCRT-III, all of which play distinct roles in the development of exosomes (28). An alternative pathway to exosome formation is the ESCRT independent pathway, and despite the different pathways, the exosomes that are released are alike in structure but vary in the cargo they carry (27).

Exosome biogenesis occurs alongside cargo packaging (**Figure 2**), with contents—proteins, lipids, and nucleic acids—reflecting the cell of origin (28). Key cargo includes RAB GTPases, ALIX, and TSG101, which are involved in membrane transport (28). RAB7, RAB11, RAB27, and RAB35 regulate exosome secretion by directing MVB trafficking and fusion with the plasma membrane (29). Tumor cells often upregulate RAB proteins to enhance exosome release, highlighting them as potential targets for cancer immunotherapy. Further research is needed on cancer-derived exosomal (CDE) RAB regulators.

In addition to regulation of exosome formation, exosome cargo also contains microRNA (miRNA) that regulate gene expression within recipient cells, and these are the highest population of RNA within exosomes (30). Exosomal miRNAs are very stable and are useful for studying exosomes (30). Under pathological conditions, tumor derived-exosome miRNAs have been found to promote lung cancer metastasis by silencing genes that down-regulate the epithelial mesenchymal transition (EMT) (31). In therapy, exosomal miRNAs are being used as tumor markers for the molecular diagnosis of tumors (32).

## Where do these exosomes go?

To facilitate intercellular communication, the exosome absorption and secretion pathways can cross paths within a cell, but the nature in which these pathways intersect varies in complexity depending on the fate of exosome cargo (33). The mechanism by which cells absorb exosomes is classified into two, one is non-specific and the other is specific uptake (34). All cells can utilize nonspecific mechanisms to absorb exosomes; however, specific uptake is necessary to allow the target cell to absorb all exosome contents relative to the host cell’s specificity with respect to cargo sorting (34). Conservation of the signature of the host cell within the exosome through conserved tropism between host and target cells promotes exosome specificity via recognition motifs that can always be recognized on these target cells by exosomes (34). An example is neuroblastoma cells where exosomes only recognize cells positive for CD63 for cargo selection (34, 35).

Upon contact with the target cell, exosomes exert their function through direct fusion with the plasma membrane or internalization within the cell (34). Direct fusion occurs when transmembrane ligands on the exosome surface bind to receptors on the surface of target cells and these trigger a signaling cascade within the cells that exert functions that may be immunomodulatory or apoptotic in nature (34).). Internalization occurs when the primary function of the target cell is to engulf the exosome followed by the release of exosome contents into the cell (34). One of the ways in which internalization is achieved is through clathrin-mediated endocytosis where the vesicles are internalized and subsequently fused with endosomes (34). As cancer cells secrete exosomes aggressively to promote tumor microenvironment (TME) activities, they can also improve exosome uptake by overexpressing of transferrin which is an essential cargo during internalization through clathrin-mediated endocytosis (34). Here we can compare how cancer cells may up-regulate exosome secretion by enhancing RAB regulatory factors and they also enhance exosome uptake by target cells via transferrin overexpression to ensure the seamless transfer of CDE cargo.

## Cancer-derived exosomes in cancer therapy

**Figure 3:**
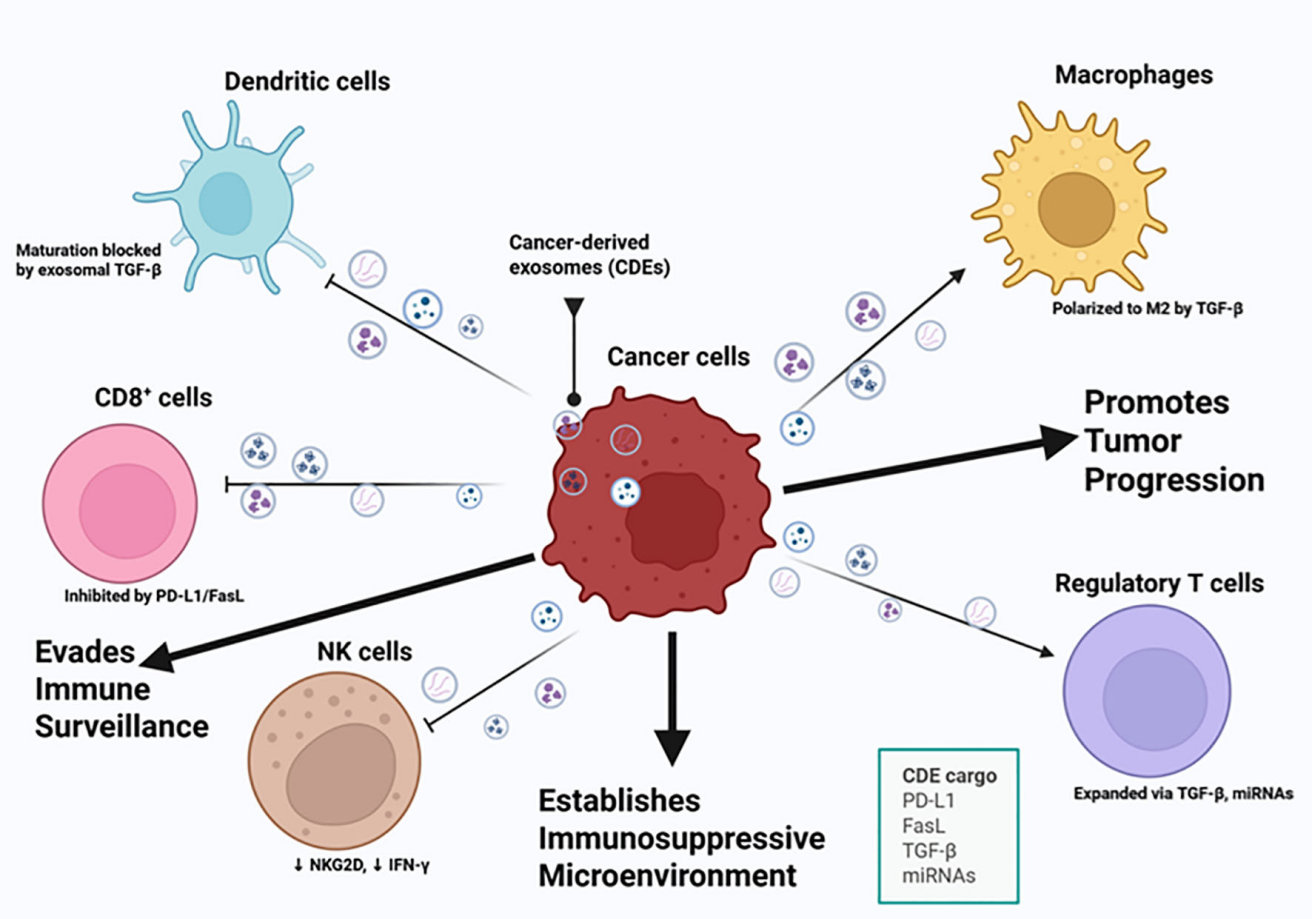
Cancer-derived exosomes mediate immune evasion and tumor progression. This illustration highlights key cancer hallmarks related to immune evasion and tumor progression, including the ability of cancer cells to avoid immune destruction, sustain proliferative signaling, induce angiogenesis, and activate invasion and metastasis. Cancer-derived exosomes (CDEs) carry immunosuppressive and oncogenic cargo, including PD-L1, FasL, TGF-β, and specific microRNAs (miRNAs), which modulate the function of key immune cell types within the tumor microenvironment. These exosomes inhibit CD8 + T cell activity through PD-L1 and FasL signaling, suppress natural killer (NK) cell cytotoxicity by downregulating NKG2D and IFN-γ, and block dendritic cell maturation via TGF-β. CDEs also promote the expansion of regulatory T cells and polarize macrophages toward an M2 phenotype, both contributing to an immunosuppressive microenvironment. This exosomal crosstalk effectively reprograms the immune microenvironment, allowing cancer cells to circumvent immune surveillance, establish an immunosuppressive niche, and promote tumor progression. Figure was designed using BioRender.com.

Cancer-derived exosomes (CDEs) are exosomes released by tumor cells in the TME and the primary way in which they regulate the TME is by altering the expression of immune cells (**Figure 3**) (36). Secondary mechanisms CDEs can employ in the TME include changing the way in which B cells, T cells, natural killer (NK) cells, and macrophages respond to the TME (36). CDEs have been studied extensively over the years, as they are key regulators of TME and may serve as potential biomarkers for diagnosis (36). Apart from regulating immune cells in the TME, CDEs can reprogram stromal cells into cells that support the formation of premetastatic niches in surrounding tissues (2). Considering the dominant control CDEs have over immune cells, the rest of the review focusses on the mechanisms by which CDEs control immune cell activity, possible crosstalk with immune cell derived-exosomes and possible therapeutic targets that can be exploited in these signaling cascades.

Building on this understanding of CDE–mediated immunosuppression, it is crucial to examine the roles of the various immune cells within the TME. Immune cells including regulatory T cells (Tregs), B cells, myeloid-derived suppressor cells (MDSCs), macrophages, dendritic cells, natural killer (NK) cells, and monocytes serve as both targets and mediators of exosome-driven signaling, shaping antitumor immunity or, conversely, contributing to immune evasion. Understanding how these immune cells interact with exosomal cargo provides a foundation for developing strategies that harness immune-derived exosomes (IDEs) to restore immune surveillance and enhance cancer immunotherapy.

## T cells

T cells, key players in the adaptive immune response, originate in the bone marrow as pro-T cells and mature in the thymus, where they become capable of protecting the host from infections and cancer (37). Immature T cells initially lack a T cell receptor (TCR) and gain antigen specificity through VDJ recombination during maturation, committing to a single antigen for their lifespan as naïve T cells (37). CD4+ T cells, known as helper cells, coordinate immune responses primarily through cytokine release and play a critical antitumor role despite limited cytotoxicity (38, 39). On the contrary, CD8+ T cells are highly cytotoxic and can induce apoptosis in cells presenting antigens recognized by their TCRs (39).

One study showed that CDEs were found to decrease IFN-γ, a critical cytokine in immune responses, in CD4+ and CD8+ T cells, as well as a decrease in Tregs that regulate immune responses by maintaining self-tolerance and exaggeration of immune responses (**Figure 3**) (40, 41). Another study showed that under an immune competence state, PDL-2 from CDEs are manipulated in a PD-1-mediated mechanism which serves to damage the integrity of T cells by upregulating Tregs and downregulating tumor-infiltrating T cells (TIL-Ts) (42).

When we focus on the study by (41), the effect of CDEs on IFN-γ and Tregs is independent of each other, however, they conjointly decrease the immune response with the TME. As Tregs naturally controls exaggerated immune responses, it does not necessarily mean that immune responses stay upregulated when Tregs is depleted as Tregs is mostly active when immune responses stay abnormally consistent above a certain threshold. This may indicate that CDEs within this context prioritize depleting IFN-γ which is more critical for immune response efforts in the TME. This may also suggest that the decrease of Tregs in the presence of CDEs is dependent on the type of cancer cells the study was using, TME conditions etc. which plays a role in the way CDEs dictate the pro-tumorigenic conditions in the TME. As opposed to the Liu et al. (43) study where CDEs were shown to directly increase Tregs to downregulate the immune response. Here we can observe that in the study (40; Hussain and Malik, 2022), the decrease in Tregs is not directly associated with cancer progression, however, in another study (42), an increase in Tregs is the major factor associated with cancer progression. This contrast in studies highlights the versatility of CDEs in their ability to manipulate a variety of immune cells and should be considered when studying their effect on T cells. Overall, these studies show how exosomes within the TME further cancer progression by promoting an immunosuppressive environment by downregulating CD4+ and CD8+ T cell function but also highlight the need for more research into counteractive measures against T cell manipulation.

## B cells

B cells support adaptive immunity alongside T cells through antigen-specific mechanisms (44). Although cancer research has traditionally focused on T cells, recent studies highlight the importance of tumor-infiltrating B cells (TIL-Bs) in enhancing T cell responses (44). TIL-Bs contribute to antitumor activity through the presentation of specialized antigens and interactions with T and NK cells, helping to transform the tumor microenvironment (TME) into a hostile space for cancer cells (45). Although the influence of tumoral exosomes on TIL-Bs remains underexplored, emerging research continues to define their role. Additionally, B cells produce antigen-specific antibodies, which generate memory cells for rapid secondary responses, and assist in directing NK and myeloid cell cytotoxicity toward tumors (44).

A group of B cells known as regulatory B cells (Bregs) has been found to support tumor immunosuppression, however, the mechanism by which they inhibit antitumor immunity in TME is still unknown (46). In a colorectal cancer (CRC) study, CDEs were shown to enhance Bregs activity by carrying long noncoding RNA (lncRNA) in their cargo (46). The IncRNA in question is known as HOTAIR, where cancer-derived HOTAIRs differentiated B cells into a regulatory phenotype associated with programmed death ligand 1 (PD-L1), these PD-L1+ B cells then inhibit the cytotoxic activity of CD8 + T cells promoting an immunosuppressive TME (46). More recently, a study done on exosomes from a murine CRC cell line shows that these CDEs prevent B cell proliferation and survival, moreover, they polarize B cells into the regulatory B cell phenotype that contributes overall to the decreased immune response toward cancer (47). The effect of CDEs from the murine CRC cell line crossed into T cell territory as they were also involved in altering the activity of CD8+ T cells (48). The results found in CRC cells show the extent to which CDEs will promote an immunosuppressive environment where they polarize immune cells into phenotypes which promote TME. These studies also highlight the need for more research on preventing immune cell polarization into phenotypes favorable for cancer progression.

## Macrophages

Macrophages play a vital role in both innate and adaptive immunity, forming the first line of defense before full immune activation (49). Their phenotype is shaped by cytokine signals: lipopolysaccharides induce the pro-inflammatory M1 type, while IL-4/IL-13 promote the anti-inflammatory M2 type (49). Among the M2 subtypes, tumor-associated macrophages (TAMs) are the most notable. Activated by A2 adenosine receptor agonists and TLR ligands, TAMs support tumor proliferation within the tumor microenvironment (TME) (M. 50). Due to its abundance and tumor-promoting role, reprogramming TAMs from the M2 to the anti-tumor M1 phenotype is a promising immunotherapeutic strategy (51). In particular, this M1/M2 polarization mirrors how B regulatory cells (Bregs) are driven into immunosuppressive phenotypes by cancer-derived exosomes (CDEs), a recurring mechanism through which CDEs manipulate various immune cells, including macrophages.

Studies have shown that CDEs in breast cancer promote macrophage M2 polarization by delivering circ-0001142, which is a circular RNA (circRNA) recently found to be highly expressed in breast cancer cells, subsequently interfering with autophagy and increasing tumor proliferation (52, 53). A defining signature of the formation of the premetastatic niche, necessary for metastasis, is the entry of immunosuppressive macrophages where CDEs polarize macrophages into the M2 phenotype distinguished by enhanced expression of PD-L1 and promoting tumor metastasis (**Figure 3**) (5). A study by Theodoraki et al. (54) shows that exosomes derived from HNSCC cells are involved in macrophage polarization into the M2 phenotype and are accompanied by increased levels of CXCL4. As recent studies continue to suggest the influence of CDEs on macrophage polarization into pro-tumorigenic phenotypes, this is a significant gap in CDE research, as more studies need to be done to counteract this mechanism and promote M1 phenotypes necessary for an anti-tumorigenic initiative.

Another study has shown that CDEs in cervical cancer delivered the TIE2 protein, involved in vascular quiescence and angiogenesis, to macrophages that promoted angiogenesis in TME (55, 56). CDEs have also been found to deliver miRNAs to macrophages in an intrahepatic cholangiocarcinoma study, such as miR-183-5p, which polarizes macrophages into the PD-L1 + phenotype, which similarly to PD-L1+ B cells, inhibits the cytotoxicity of CD8+ T cells promoting an immunosuppressive environment (46, 57). A combined initiative of B cell and macrophage immunotherapy initiative has the potential to prevent polarization into immunosuppressive phenotypes, and this is more effective than individual immune cell immunotherapies.

## Dendritic cells

Dendritic cells (DCs), key antigen-presenting cells in conjunction with macrophages and B cells, bridge innate and adaptive immunity (58). They exist in immature and mature forms. Immature DCs, found on mucosal surfaces, express low MHC levels but are antigen processing and migratory. Mature DCs have reduced antigen processing but enhanced migration (58). Using pathogen recognition receptors (PRRs), DCs detect PAMPs or DAMPs, internalize antigens, and present them via MHC to T cells (58). Beyond pathogens, DCs also process tumor antigens. Reduced DC levels in cancer suggest tumor-driven suppression of DC function within the tumor microenvironment (59).

Studies have shown that CDEs promote immunosuppressive TME by suppressing DC maturation and activity (**Figure 3**) (42). With the loss of DC function, tumor antigens cannot be processed and presented to T cells that contribute to cancer cell proliferation (42). DC differentiation is directly related to MDSC expression levels to the extent that loss of function of MDSC directly affects DC maturation (42). CDEs inhibit DC differentiation by interfering with myeloid cells, and employ molecules such as prostaglandin E2 (PGE2), TGF-β and heat shock proteins (42). CDEs derived from prostate cancer were found to prevent DC differentiation leading to accumulation of their MDSC precursors known to be involved in suppressing the immune response (60). Another study shows that exosomes acquired from the cerebrospinal fluid (CSF) of glioblastoma multiforme (GBM), one of the most aggressive and common brain tumors, contained Galectin-9, which is a molecule involved in preventing DC cell maturation (42, 61).

As DCs are crucial for antigen presentation, CDEs ensure their inactivity, lowering the frequency of immune responses in the TME. Until this point, it is evident that cancer immunotherapy should not only be directed towards only a subset of immune cells and rather all immune cells as CDEs employ a variety of mechanisms to promote immunosuppressive TMEs. Upregulating a subset of immune cells in the TME during cancer immunotherapy does not necessarily solve the problem, as CDEs focus their efforts on down-regulating a different subset of immune cells and this highlights the complexities of developing a therapeutic strategy to counteract CDEs. Like the suggestion of a combined B-cell and Macrophage immunotherapy, there should also be a combined DC and MDSC immunotherapy approach, as there is a correlation between DC differentiation and MDSCs which has the potential to produce greater therapeutic effects in cancer immunotherapy.

## Myeloid-derived suppressor cells

Myeloid-derived suppressor cells (MDSCs) inhibit both innate and adaptive immunity and are heterogeneous in transcriptional activity and differentiation states (62, 63). Under pathological conditions such as cancer, MDSCs resemble neutrophils or monocytes but deviate from their normal immune functions to promote tumor progression (64). Like Tregs, MDSCs regulate immune responses, but their suppressive functions are amplified in cancer and chronic inflammation (63). Pro-inflammatory cytokines such as PGE2 and TGF-β hinder DC maturation and promote MDSC differentiation, contributing to immune evasion (42). CDEs also alter DC development and increase MDSC accumulation, leading to localized immunosuppression in the TME (60). Targeting DC differentiation may offer a strategy to reduce MDSC-mediated suppression and restore immune competence.

When we shift the focus to MDSCs-derived exosomes derived in the TME, it was observed that these exosomes promoted the development of castration-resistant prostate cancer by upregulating the S100A9/circM1D1/miR-506-3p axis (65). S100A9 is a calcium binding protein that is said to have implications in cancer associated with inflammation, circM1D1 expression is highly upregulated in prostate cancer cells treated with MDSC exosomes and miR-506-3p was found to be an inhibitor of CRC progression through EZH2-targeted mechanisms (65–67). MDSC exosomes in this study were associated with faster progression, migration, and invasion of prostate cancer cells (65). In a concurrent experiment, they observed that circM1D1 downregulated MDSC exosome-mediated prostate cancer progression, and S100A9 from MDSC exosomal cargo was able to convert circM1D1 expression to sponge miR-506-3p, masking its antitumoral effects and effectively promoting prostate cancer cell progression (65). This demonstrates that the promotion of tumor progression in the MDSC context can occur in two ways, which are through CDE mediated mechanisms and through MDSC exosomal mechanisms. Immunotherapy would have to be targeted at the regulators of each pathway such as HSP70 or the S100A9/circM1D1/miR-506-3p axis, however, targeting CDEs may produce more promising results, as they inhibit the activity of MDSCs before they even reach a stage of producing pro-tumorigenic exosomes.

Studies done on CDEs of renal cancer have shown that MDSC-mediated immunosuppression in TME is achieved through antigen-specific mechanisms and is highly dependent on the presence of HSP70 as a regulatory factor (68). These findings have potential in the therapeutic landscape by actively blocking MDSC activity or preventing the expression of HSP70 (42, 68). Furthermore, a study carried out on highly metastatic colorectal cancer cells shows that CDEs contain lncRNA MIR181A1HG which promotes liver metastasis through MDSC recruitment and is also a key player in extracellular matrix remodeling (69). As the primary mechanism used by CDEs for cancer proliferation is through MDSC recruitment, more strategies aimed at halting CDE-mediated MDSC recruitment must be studied to bridge this research gap as the only strategy available to date is targeting DC differentiation which is still in development.

## Natural killer cells

Natural killer (NK) cells are innate lymphocytes involved in antitumor and antiviral responses (70). Their activation depends on signals from activating or inhibitory receptors, allowing them to distinguish self from nonself through recognition of MHC I (70, 71). Once activated, NK cells kill compromised cells by releasing cytotoxic granules that induce apoptosis (70). However, in cancer, a subset called dysfunctional NK cells fails to eliminate malignant cells due to the immunosuppressive tumor microenvironment (TME) (72). The TME alters NK function by disrupting activating signals, enhancing inhibitory pathways, and interfering with metabolism. Restoring NK activity by targeting these disruptions is a key focus of cancer immunotherapy.

A study was conducted in CDEs from samples of hepatocellular carcinoma (HCC) adjacent to NK cell function where qRT-PCR was used to identify circular ubiquitin similar to PHD and ring finger domain 1 RNA (circUHRF1) in HCC CDEs (73). circUHRF1 in the HCC CDEs cargo was found to promote immunosuppression in the TME by contributing NK cell dysfunction in HCC (73). The mechanism by which circUHRF1 acts is by promoting TIM-3 expression, which is involved in T cell exhaustion during cancer, and downregulates miR-449C-5p, which is a gene silencer for the Tim-3 gene (73, 74). circUHRF1 is not only involved in TME regulation, as it also presents a challenge in cancer immunotherapy by resisting anti-PD1 therapy (73). So far, CDEs have portrayed a variety of mechanisms to counteract immune cell function, showing that they are the focal point of immunosuppressive efforts by tumor cells in the TME.

A study carried out on CDEs derived from oral cancer (OC) cell lines shows an elevation of TGF-β via mass spectrometry analysis of protein cargo of these exosomes (75). In OC studies, TGF-β is involved in inhibiting NK function in OC samples (42, 75). The enrichment of TGF-β coincides with the inhibition of key NK cell receptors such as NKG2D and NKp30, however, the hypothesis suggests that the deeper lying mechanisms need to be studied (75). It is evident that targeting TGF-β in cancer immunotherapy has the potential to restore the function of DCs and NK cells within the TME. Flow cytometry analysis of OC CDEs together with NK cells further revealed the gradual decrease over a week in killer cell lectin-like receptor k1 (KLR-K1) and the natural cytotoxicity triggering receptor 3 (NCR-3) (75). KLR-K1 is a critical receptor in immune cells that promotes an antitumor effect against cancer, while NCR-3 is responsible for NK cell identification as well as destruction of target cells (76, 77). The study on OC-derived CDEs reveals that CDEs gradually suppress natural killer (NK) cell function rather than causing immediate inhibition. This temporary lag phase presents a potential window for therapeutic intervention to prevent NK cell suppression in cancer immunotherapy.

## Monocytes

Monocytes, derived from the bone marrow, are key components of the innate immune system (78). In cancer, they act as critical regulators, capable of both pro- and anti-tumorigenic functions (79). They typically accumulate early during tumor development and metastasis. While monocytes can induce tumor cell apoptosis through cytokine release and phagocytosis, this has mainly been observed *in vitro*, with *in vivo* relevance still unclear (78, 79). Monocytes can differentiate into tumor-associated macrophages (TAMs) or suppress T cell activity, aiding tumor immune evasion. Their dysfunction in cancer highlights the need for therapies that target monocyte-driven tumor progression.

CDEs in colorectal cancer have been shown to interfere with monocyte differentiation into macrophages, limiting tumor antigen presentation to the immune system (80). When monocytes merge their membranes with CDEs, this alters their phenotype into a phenotype that does not express the human leukocyte antigen-DR (HLA-DR), its costimulatory molecule, and only expresses a surface marker CD14 (81). This is significant as the altered monocyte phenotype forms an integral mediator in tumor immunosuppression in the TME and other mechanisms CDEs employ to interrupt monocyte differentiation include disrupting the STAT3 signaling cascade and promoting the formation of reactive oxygen species (ROS) (82). The disruption of monocyte differentiation by CDEs builds on previous discussions about macrophages, where CDEs primarily downregulate immune cells that induce a domino effect on the function of adjacent immune cells targeted toward the TME region. This means that therapeutic efforts can be directed at the source of the domino effect rather than only a single immune cell to ensure that all immune cells are effective against cancer cells.

To better illustrate their role in shaping the tumor immune microenvironment, **Table 1** summarizes the major cargos carried by CDEs and their downstream effects on immune targets involved in tumor immunosuppression.

**Table 1:** Summary of CDE cargos and their downstream effects on immune targets in tumor immunosuppression.

CDE cargo	Immune target	Mechanism of action	Effect	Reference
PDL-2	T cells	PD-1 mediated; Tregs upregulated and TIL-Ts downregulated	Damages integrity of T cells	(42)
lncRNA (HOTAIR)	B cells	Promotes the polarization of B cells into PDL-1 mediated Bregs phenotype	Diminishes cytotoxic activity of CD8+ T cells	(46)
circRNA (circ-0001142)	Macrophages	Polarize Macrophages into M2 phenotype	Interferes with autophagy and promotes tumor proliferation	(52, 53)
CXCL4	Macrophages	Polarize Macrophages into M2 phenotype	Promotes tumor proliferation	(54)
TIE2	Macrophages	Active in the presence of VEGF-A and Angiopoietin in TME	Promotes angiogenesis in the TME	(55, 56, 83)
miR-183-5p	Macrophages	Polarizes macrophages into PDL-1+ phenotype; Transported by M2 TAM regulated Akt/NF-KP pathway	Inhibits cytotoxic activity of CD8+ T cells; Accelerates cancer progression	(46, 57, 84)
TGF-β, PGE2 and heat shock proteins	Dendritic cells	Interfere with myeloid cells which downregulate DC differentiation	Leads to loss of DC driven tumor suppression	(60).
Galectin 9	Dendritic cells	Prevents DC cell maturation via Gal-9/Tim-3 signaling	Leads to loss of DC driven tumor suppression	(42, 61, 85)
circUHRF1	Natural Killer cells	Inhibits miR-449C-5p which is responsible for silencing Tim-3 gene. Upregulation of Tim-3 disrupts NK activity	Loss of NK cell contributes to tumor immunosuppression	(73, 74)
lncRNA MIR181A1HG	Myeloid Derived Suppressor cells	Promotes upregulation of MDSC	Metastasis, ECM remodeling and tumor immunosuppression	(69)

These examples underscore how CDE cargos actively remodel the immune landscape, setting the stage for therapeutic strategies aimed at disrupting exosome-mediated immunosuppression. In contrast, immune-derived exosomes (IDEs), such as those secreted by dendritic cells or activated T cells, can be engineered to carry immunostimulatory molecules, tumor antigens, or checkpoint inhibitors to activate the immune system against cancer (86). These therapeutic IDEs offer the potential to reverse the immunosuppressive tumor microenvironment, enhance antigen presentation, and stimulate robust adaptive immune responses.

## Therapeutic potential of immune-derived exosomes

**Figure 4:**
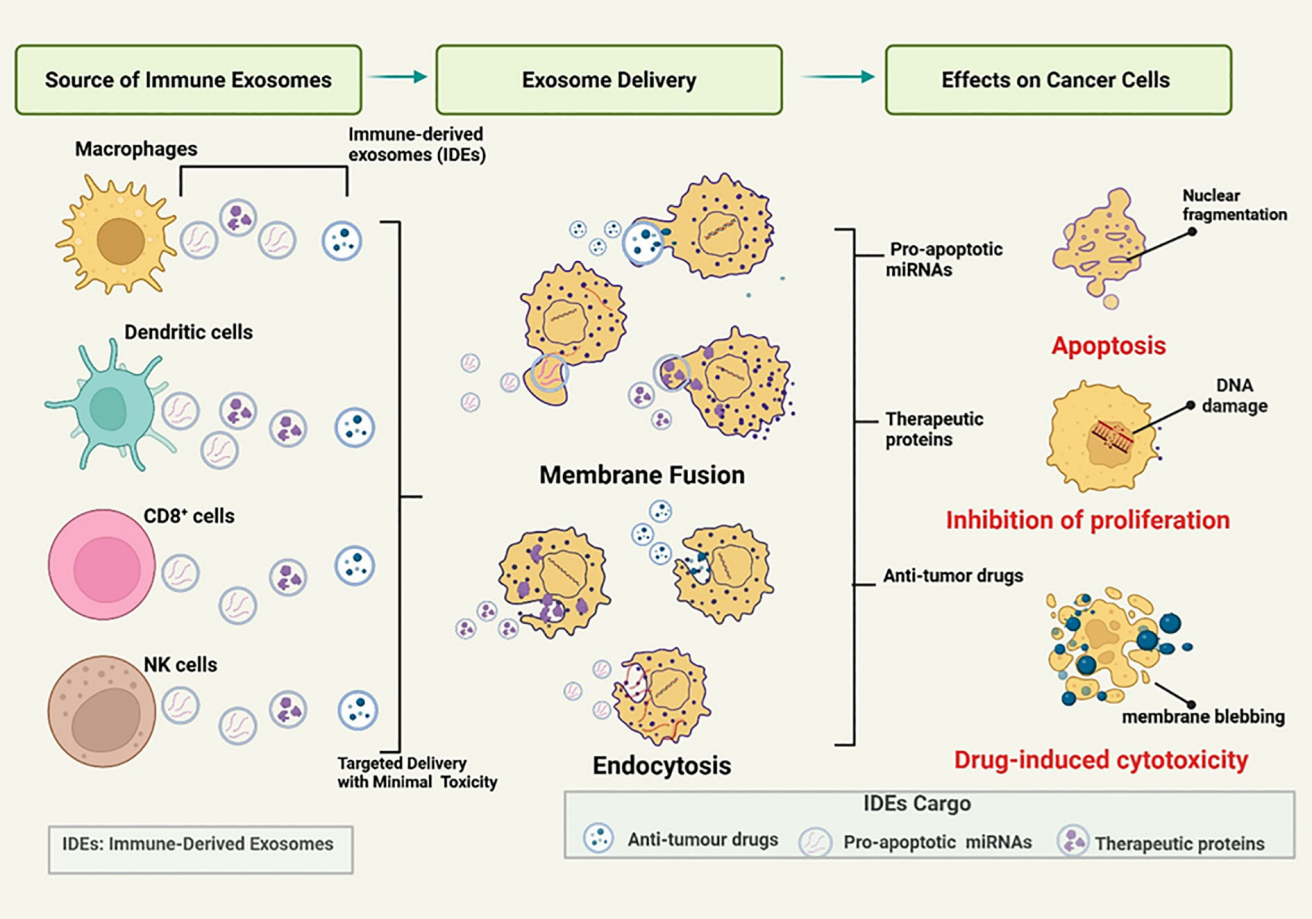
Mechanisms by which immune-derived exosomes (IDEs) mediate therapeutic effects. Immune cells - including macrophages, dendritic cells, CD8 + T cells, and natural killer (NK) cells - release immune-derived exosomes (IDEs) loaded with therapeutic cargo such as pro-apoptotic microRNAs, antitumor drugs, and therapeutic proteins. These IDEs are taken up by cancer cells through membrane fusion or endocytosis, enabling targeted delivery with minimal systemic toxicity. Upon delivery, the cargo induces distinct anticancer effects: (1) apoptosis, driven by pro-apoptotic miRNAs and characterized by nuclear fragmentation; (2) inhibition of proliferation, mediated by therapeutic proteins that cause DNA damage and cell cycle arrest; and (3) drug-induced cytotoxicity, where antitumor drugs trigger membrane blebbing and cell death. Collectively, IDEs represent a multifunctional platform that integrates immune surveillance with targeted therapeutic action against cancer cells. Figure was designed using BioRender.com.

Strategies targeting crosstalk between CDEs and immune cells aim to reverse immunosuppression within the TME. Exosomes are promising as diagnostic biomarkers, drug delivery vehicles, and therapeutic targets in cancer immunotherapy (87). While CDEs often promote immunosuppression, IDEs, such as those secreted by dendritic cells or activated T cells, can be harnessed as therapeutic agents (86). IDEs can be engineered to deliver tumor antigens or immunostimulatory molecules, activating adaptive immunity and counteracting the immunosuppressive effects of CDEs. Given that the immune balance is shaped by this exosomal interplay, increasing the function of the IDEs could restore immunocompetence and counteract the hallmarks of cancer. **Figure 4** illustrates the mechanisms by which IDEs exert therapeutic effects. IDEs, secreted by immune cells such as dendritic cells, macrophages, CD8^+^ T cells, and NK cells, deliver pro-apoptotic miRNAs, antitumor drugs, and therapeutic proteins to cancer cells, inducing apoptosis, inhibiting proliferation, and triggering cytotoxicity with minimal systemic toxicity (88).

## B cell-derived exosomes

B cell-derived exosomes (BDEs) are released by B cells and have been found to carry an MHC-II molecule conjugated with a peptide (pMHC-II) (89). This pMHC-II is only released by BDEs upon B cell activation so that helper T cells can initiate their immune response to that antigen (89). BDEs have potential as therapeutic drug carriers when it was shown that they can carry miR-155 in mouse models (89). In the context of cancer regulation, plasma cell-derived exosomes which are derivatives of B cells regulate tumor proliferation by carrying miR-330-3p which downregulates TPX2; a critical gene involved in sustaining melanoma cell proliferation (89). Protocols surrounding down-regulation of TPX2 through BDEs have not been fully optimized and need to be validated, however, they show great potential to inhibit the development of melanoma (89). In another study, BDEs were treated with zinc oxide nanocrystals (ZnNCs) and these promoted cytotoxicity against Burkitt lymphoma (90). These BDEs were further modified by adding an anti-CD20 monoclonal antibody to promote lymphoma cell specificity (90). However, BDEs have great potential in cancer immunotherapy, because of the limited number of studies they have not been fully characterized in this context (90). Modifications of BDEs show great promise regardless, as observed with results obtained from Burkitt lymphoma studies (90).

## T cell-derived exosomes

T cell-derived exosomes (TDEs) are released by T cells and characterized according to the functions of parent T cells such as cytotoxic effects, regulation of antibody release by B cells, specificity against antigens and mediating cytokine release (91). TDEs regulate immune responses by coordinating the activity of other immune cells in mediating APCs (91). Considering that T cells are divided into CD8 +, CD4 + and Tregs, each subset releases their own exosomes which have their own distinct functions (91). Multiple studies have shown that CD8 + TDEs control information transfer between immune cells and tumor cells (91). These CD8 + TDEs promote T cell cytotoxicity which subsequently destroys tumor cells (91). A study has shown that CD8 + TDEs have increased programmed cell death- 1 (PD-1) expression which promotes toxicity by binding to PD-L1 and downregulating PD-L1 induced suppression of cytotoxic T cells (91). In addition to mediating information exchange between tumor cells and immune cells, CD8+ TDEs are also involved in halting tumor progression (91). Another study has shown that CD8 + TDEs without CD45RO carry miR-765 which is involved in inhibiting estrogen-driven development of uterine corpus endometrial cancer (UCEC) (91). Another way that CD8 + TDEs can down-regulate tumor proliferation is by depleting supporting mesenchymal tumor stromal cells (MTSCs) (91). CD8 + TDEs are not only involved in antitumor responses and can also be protumor, making therapeutic avenues around TDEs more complex (91). Exosomes from spent CD8 + T cells disrupt the production of crucial antitumorigenic cytokines such as IFN-γ, IL-2 and this causes CD8 + T cells to lose their cytotoxic abilities in antitumorigenic responses (91).

CD4 + TDEs promote antitumor responses by mediating crosstalk between CD4 + T cells and other important immune cells such as macrophages, NK cells, and CD8 + T cells (91). CD4 + TDEs carry miR-25-3p, miR-155-5p, miR-215-5p and miR-375 which promote CD8+ T cell-mediated antitumorigenic responses (91). CD4 + TDEs initiate these antitumor responses without provoking Tregs immune regulation (91). Tregs on the other hand, contrary to their other T cell counterparts, are more involved in immunosuppressive activity and are usually more pronounced in the TME (91). In a HNSCC study, patients received a variety of chemotherapeutic drugs such as cetuximab and ipilimumab, and Tregs-derived exosome expression was monitored (91). It was found that Tregs-derived exosome expression was increasing from its standard levels, indicating that Tregs-derived exosomes may serve as biomarkers in HNSCC (91). It can therefore be understood that up-regulation of factors that promote T cell derived exosome secretion can be promising for cancer immunotherapy, which negates g the effects of CDEs in the TME. It is evident that under pro-tumorigenic conditions, the balance needs to be shifted in favor of T cell derived exosome secretion to activated T cells which had their functions impaired by CDEs.

## Macrophage-derived exosomes

Previously we mentioned that CDEs employ mechanisms to convert anti-tumorigenic macrophages M1 like into the more aggressive pro-tumorigenic M2 like phenotype known as TAMs and this can be manipulated in a therapeutic context in the reverse to promote more M1 like phenotypes through macrophage-derived exosomes (MDEs) (92). The first strategy to promote M1 phenotypes is to target and prevent TAM formation, and this can be done using a variety of mechanisms (92). The first mechanism is to block macrophage recruitment for pro-tumorigenic purposes, and this is done using inhibitors such as vascular endothelial growth factor (VEGF) or colony stimulating factor (**Figure 4**) (92). The second mechanism is by reducing the number of TAMs in the TME and many studies have used liposomal chondrates that reduce the vasculature in this region, preventing adequate blood supply to the TAMs (92). The third mechanism is to condition TAMs to a more favorable M1-like phenotype, and this can be achieved using cytokines such as IL-12 or M2 inhibitors such as miR-125b (92). Another mechanism involves the inhibition of the CD47-SIRPα pathway for advanced macrophage cell phagocytosis (92). Considering that CD47 is a marker that is highly expressed in cancer cells and interacts with SIRPα to prevent their own phagocytosis, this pathway can be inhibited through anti-CD47 or anti-SIRPα therapy leading to more phagocytosis of cancer cells (92).

In the case of MDEs, these can be engineered into the M1 like phenotype as they inherit their characteristic traits from macrophages and may serve as anticancer drug vehicles (92). These MDEs were modified with aminoethyl anisamide (AA), which binds to the α receptors in lung cancer and plays a role in stopping pulmonary metastasis of nonsmall cell lung cancer (92). A study was carried out in macrophage-derived M1 exosomes where these exosomes were polarized into the M1 phenotype with the aid of M1 enhancers such as NF-KB p50 siRNA, which silences the antiapoptotic activity of NF-KB-P50 in cancer cells, and miR-511-3p (93, 94). The surface of these M1 MDEs was also lined with IL4R-Pep1 so that they can bind to the IL4R receptors of TAMs (94). It was found that these TAMs took up these M1 MDE binding peptides and downregulated essential M2 macrophage genes that ultimately promoted the expression of M1 markers while downregulating M2 markers (94). Modifying these M1 MDEs contributed to stopping tumor growth, preventing the expression of key M2 cytokines while concurrently promoting the expression of M1 cytokines (94). M2 reprogramming using M1 MDEs is a promising strategy in cancer immunotherapy, as the global decrease of TAMs in the TME means that certain cancer hallmarks cannot be sustained as the immune response is in favor of immunocompetence rather than immunosuppression. Halting the activity of TAMs may indicate that other immune cells will follow suit with M1 macrophages considering the proximity of their crosstalk and more studies need to be done to ensure the maintenance of the M1 phenotype in cancer immunotherapy.

## Natural killer cell-derived exosomes

NK cell-derived exosomes (NKDEs) are derivatives of NK cells and can perform signature NK cell functions according to the signal from NK activation or NK inhibitory receptors (95). When NK cells are stimulated to kill cancer cells, NK cells release NKDEs that perform antitumorigenic activities by releasing cytotoxic molecules such as perforin, granzymes, and miRNAs (**Figure 4**) (95, 96). NKDEs show great potential as enforcers of immune modulation and cancer immunotherapy due to their intrinsically latent antitumor influence (96). Therefore, it can therefore be assumed that NKDEs activity is silenced under pro-tumorigenic conditions, as parent NK cells have little function under these conditions (95). However, since this is a two-way road in terms of cancer immunotherapy against CDEs, studies have found ways to use NKDEs to deliver therapeutic drugs against cancer and the activation of NK-activated responses to promote cytotoxicity.

A recent study of triple negative breast cancer exploited the cargo carrying ability of NKDEs to determine whether they could deliver Sorafenib, an antitumor drug, to these cancer cells (97). The study wanted to compare Sorafenib administration with NKDEs versus without NKDEs and it was found the administration of Sorafenib with NKDEs significantly increased the cytotoxicity towards triple negative breast cancer spheroids (*in vitro* tumor mimics), highlighting the promising potential of NKDEs in cancer immunotherapy (97, 98). In a study conducted on NKDEs loaded with oxaliplatin, NKDEs were confirmed to have benefits such as inherent inhibition of tumor growth and their ability to enhance the antineoplastic activity of oxaliplatin in CRC therapy (99). Recent studies around NKDE cancer immunotherapy focus on increasing the apoptosis inducing ability of NKDEs, as they are more potent than other techniques. The delivery of chemotherapeutic drugs such as sorafenib using NKDEs may have increased specificity for tumor cells and reduce side effects of chemotherapy, making it a promising avenue for cancer immunotherapy.

## Dendritic cell-derived exosomes

Dendritic cell-derived exosomes (DDEs) are vesicles released by DCs and possess the phenotypic characteristics of DCs which include the MHC complex, costimulatory components, and other surface markers required for communication with other immune cells (100). DDEs have more potential in tumor rejection using immune cells than traditional DC immunotherapy methods (100). DDE immunotherapy is more effective than DCs as they can maintain DC immunostimulatory characteristics without degrading and the stability of their membranes provides increased frozen storage for up to 6 months (100). DDEs possess both types of MHC molecules: MHC-I and MHC-II; and they can stimulate both helper T cell activity as well as cytotoxic T cell activity (100). The most abundant proteins in DDEs are the EGF factor 8 (MFG-E8) milk fat globule, which increases target cell exosome uptake (100).

What separates DDEs from exosomes from other immune cells is their enhanced antigen-presenting abilities, however, DCs produce greater T-cell responses (100). Some mechanisms by DDEs that stimulate antigen presentation to T cells include binding of APCs and they transfer their MHC/peptide complex to the APC, removing the need for any antigen processing (100). Another mechanism involves DDE-mediated tumor manipulation in adenocarcinoma cells that reactivate primed T cells and produce an IFN-γ mediated T cell response (100). The ability of DDEs to weaponize tumors to promote immunocompetence indicates that DDEs show great promise in cancer immunotherapy by coordinating T-cell responses against cancer cells.

A recent study produced a nano vaccine platform using DDEs and patient-specific neoantigens for personalized cancer immunotherapies (J. 49). The nano vaccine was designed for efficient cargo loading and increased cargo transportation times to lymph nodes which led to antigen specific B and T cell responses that had beneficial biosafety as well as biocompatibility (J. 49). The use of this nano vaccine system was found to significantly oppose tumor proliferation, had longer survival times, slowed down tumor incidence and eradicated lung metastasis in certain cancer models (49). The introduction of personalized DDE nano vaccine platforms provides a significant advantage in cancer immunotherapy as this eliminates the reliance on cell-based immunotherapy which is less efficient and has lower biocompatibility. In a study done by Safaei et al. (101), exosomes derived from triple negative breast cancer cells (TNBCC) could induce immunogenicity and this meant that they could improve DC vaccine immunotherapy for cancer patients.

These personalized nano vaccine systems provide a powerful avenue in DDE based immunotherapy to effectively deliver molecules which coordinate T cell responses against cancer cells as they overcome the barrier of biosafety and biocompatibility, which were major issues in DC based immunotherapy. This immunotherapy combined with the immunotherapy of other immune cell derived exosomes may pave the way for chemotherapy free cancer treatments which are mostly non-invasive.

Despite their promise, the clinical efficacy of IDE-based therapies remains limited in solid tumors compared to hematologic malignancies. This challenge arises from the hypoxic and immunosuppressive TME, which impairs T cell activity, remodels the extracellular matrix and vasculature, and drives immune suppression through Tregs, MDSCs, and TAMs (87). Addressing these barriers will be essential for unlocking the full therapeutic potential of immune-derived exosomes. In the following section, we provide a comparative overview of CDEs and IDEs, highlighting their contrasting roles in tumor progression and immune activation.

## Dual faces of exosomes in cancer: drivers of immunosuppression and agents of immunotherapy

**Figure 5:**
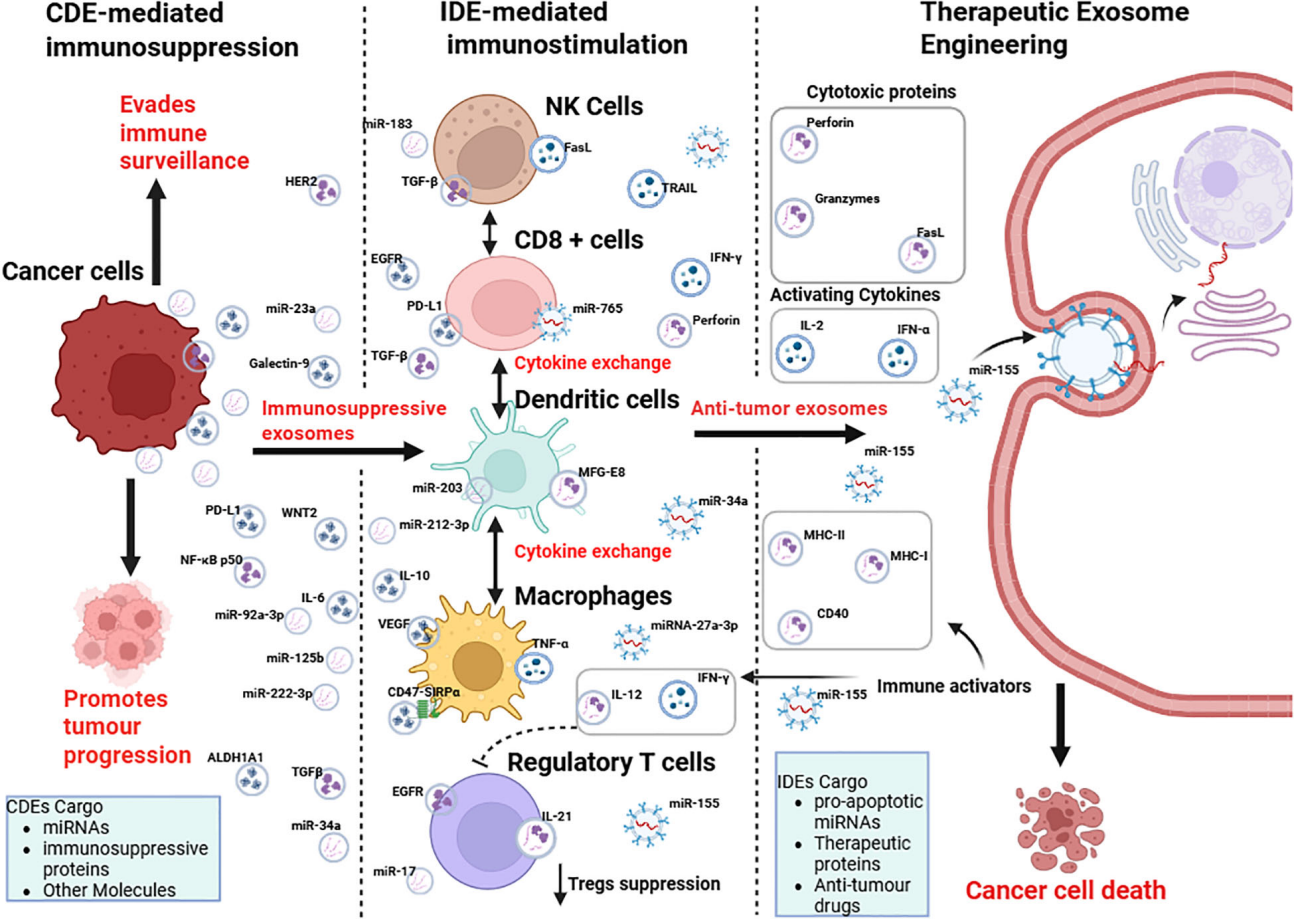
Crosstalk between cancer cells and the immune system via cancer-derived exosomes (CDEs) and immune cell-derived exosomes (IDEs), and their potential for therapeutic exosome engineering. Cancer cells release immunosuppressive exosomes (CDEs) containing miRNAs, immunosuppressive proteins, and other molecules, which promote immune evasion and tumor progression by modulating NK cells, CD8^+^ T cells, dendritic cells, macrophages, and regulatory T cells. In contrast, immune cell-derived exosomes (IDEs) carry pro-apoptotic miRNAs, cytokines, and cytotoxic proteins that stimulate anti-tumor immunity. Therapeutic exosome engineering aims to exploit IDE cargo (e.g., perforin, granzymes, IL-2, IFN-α, miR-155) to deliver immune activators and anti-tumor drugs, ultimately inducing cancer cell death. Figure was designed using BioRender.com.

Exosomes serve as critical mediators of intercellular communication within the tumor microenvironment, exerting dual functions by either suppressing or stimulating immune responses, and offering opportunities for therapeutic engineering (**Figure 5**). CDEs carry immunosuppressive and oncogenic cargo such as PD-L1, FasL, TGF-β, and specific microRNAs, which suppress CD8^+^ T cell cytotoxicity, impair natural killer (NK) cell activity, and block dendritic cell maturation (81). In addition, they encourage the expansion of regulatory T cells and direct macrophages towards an M2 phenotype, thus strengthening an immunosuppressive TME that supports tumor growth, angiogenesis, invasion, and metastasis (57). This capacity of CDEs to alter immune cell function underscores their pivotal role in tumor immune evasion.

In contrast, IDEs secreted by NK cells, CD8^+^ T cells, dendritic cells, and macrophages offer an immunostimulatory counterbalance. These vesicles are enriched with cytotoxic proteins such as perforin and granzymes, cytokines like IFN-γ, and pro-apoptotic microRNAs that restore immune surveillance and trigger cancer cell death (88). By leveraging these properties, bioengineered exosomes can be tailored to transport tumor antigens, checkpoint inhibitors, or therapeutic drugs, facilitating accurate delivery and reducing systemic toxicity (102). By shifting the emphasis from CDE-driven immunosuppression to IDE-mediated immune activation, therapeutic exosome engineering has the potential to transcend the shortcomings of existing immunotherapies for solid tumors and pave the way for a novel era of precision cancer treatments.

Thus, understanding and harnessing the opposing functions of CDEs and IDEs provides a strategic avenue for developing next-generation exosome-based therapies that precisely modulate the tumor-immune interface. Exosome-based strategies demonstrate how leveraging the immune system can be effective against cancer. Similarly, other immunotherapy methods, including immune checkpoint inhibitors, CAR-T cell therapy, and cancer vaccines, seek to restore or boost antitumor immunity. Each of these approaches operates through unique mechanisms and comes with its own set of benefits and obstacles.

## Other cutting edge cancer immunotherapies

Immune checkpoint inhibitors (ICIs) are one of the latest cancer immunotherapies which steer away from the conventional chemotherapy treatments and are being used to treat a variety of solid and liquid tumors (103). ICIs primarily act on T cells by removing any form of suppression of T cell activity from cancer cells, and this increases the cytotoxicity and antitumorigenic potential of T cells as well as other immune cells (103). The most prominent immune checkpoint pathway that cancer cells exploit to evade the immune system is the PD-1/PD-L1 pathway and ICIs, by blocking PD-1 or PD-L1 to prevent immune evasion (104). The goal of anti-PD1 or anti-PDL1 treatment is to activate cytotoxic T cells within the TME by forming a blockade between the immunosuppressive PD-1/PD-L1 ligand receptor complex (104). Immune checkpoint immunotherapy should be combined with engineered immune cell-derived exosomes to ensure global activation of cytotoxic T cells and NK cells to combat cancer. The combination of a variety of cancer immunotherapies may increase the specificity against a variety of cancer types, however, these treatment options may be costly, which is a challenge.

Chimeric antigen receptor T cell (CAR-T cells) therapy is an exciting avenue in cancer immunotherapy which has been successful in a variety of hematological malignancies (105). CAR-T cell therapy weaponizes T cells to bind tumors with overexpressed surface antigens (105). The T cells are modified with CAR which increases the specificity of T cells towards tumor surface antigens (105). Despite the FDA approval of six CAR-T cell therapies, there are still ongoing clinical trials on other diseases, and as with other cutting edge cancer immunotherapies, they present some dangerous side effects such as immune effector cell associated neurotoxicity syndrome (ICANS) (105). Considering that the goal of CAR-T therapy is essentially to arm T cells with the firepower to destroy cancer cells, these can be combined with loading of TDEs with cargo that increases T cell cytotoxicity.

In addition to established immunotherapies, recent studies indicate that cancer exosomes undergo notable transformations after treatment, affecting immune reactions and resistance to therapy (106). Grasping these post-treatment changes in exosomes is essential for enhancing immunotherapy results and addressing tactics for tumor evasion.

## Post-therapeutic modulation of cancer exosomes: implications for immunity and resistance

Emerging evidence suggests that cancer treatments, including chemotherapy and radiotherapy, can profoundly reshape the composition and function of tumor-derived exosomes. These post-therapeutic changes influence immune responses, contribute to therapy resistance, and impact clinical outcomes. For instance, chemotherapeutic agents such as carboplatin, paclitaxel, and irinotecan have been shown to markedly increase exosome release from HepG2 hepatocellular carcinoma cells, as measured by acetylcholinesterase activity assays (13). Exosome production in CAG human cells increased significantly 16 hours after treatment with melphalan, bortezomib, and carfilzomib, as measured by nanoparticle tracking analysis (11). Similarly, after paclitaxel treatment, an increase in exosome release was observed compared to untreated cells in MDA-MB-231 breast cancer cells (12). However, contradictions emerged when comparing these *in vitro* studies with ex vivo studies. A notable decrease in exosomal protein levels was reported in patients with acute myeloid leukemia (AML) after chemotherapy (14). Similarly, exosomal protein levels decreased in patients with head and neck cancer after oncological treatment (15). These discrepancies may be attributed to differences in exosome clearance, tumor burden, systemic immune responses, and technical variability between controlled *in vitro* conditions and the complex physiological environment represented in ex vivo patient samples.

Furthermore, after radiation therapy, exosomes derived from breast cancer cells (MCF7, SKBR3, and MDA-MB-231) irradiated with 2 Gy exhibited altered molecular profiles compared to controlled group without radiation (107). While these changes did not influence cell viability or radioresistance, irradiated exosomes increased migratory and invasive potential, in part through β-catenin downregulation—and were more readily internalized by endothelial cells, contributing to reduced expression of CD31 and vascular disruption. Pszczółkowska (2022) (108) reported a dose-dependent decrease in exosome concentration in both PC3 and DU145 prostate cancer cell lines after alpha radiation, although the reduction was not statistically significant. Furthermore, more radio-resistant DU145 cells secreted fewer exosomes than radio-sensitive PC3 cells. In addition, exosomes released by irradiated head and neck cancer cells induced DNA damage and replication stress in naïve recipient cells, evidenced by increased γH2A.X foci and activation of ATM/ATR kinases (109). These effects, which occur even before full exosome internalization, suggest a receptor-mediated bystander mechanism driven by radiation-altered exosomal signaling.

P-gp and other key ATP-binding cassette transporters linked to multidrug resistance are frequently present on exosome membranes (110). Exosomes can transfer P-gp from resistant to sensitive tumor cells, promoting drug resistance (111). In addition to ABC transporters such as P-gp, exosomes from resistant cancer cells also carry detoxifying enzymes such as glutathione S-transferases (GSTs), which neutralize reactive oxygen species and toxic metabolites generated by chemotherapy, thus reducing treatment efficacy (112). Furthermore, tumor-derived exosomes may also carry PD-L1, which can inhibit T cell activation and contribute to immune evasion by suppressing the antitumor immune response (113). In another study by Theodoraki et al. (9), exosomal PD-L1 was reported to be the earliest indicator of failure in treatment in patients with Head and neck cancer. These studies highlight the potential of exosome profiling as a noninvasive biomarker for predicting therapeutic response and guiding early intervention to prevent recurrence.

## Cancer-derived exosomes: biomarkers of immune status and tools for diagnosis, therapy monitoring, and treatment

Exosomes have emerged as promising non-invasive biomarkers because of their stability in body fluids and their molecular cargo reflective of the tumor microenvironment. Their diagnostic, prognostic, and predictive potential is particularly evident in immunotherapy, where PD-L1+ exosomes have shown utility as biomarkers for immune checkpoint inhibitor (ICI) response and resistance (10). In the KEYNOTE-028 trial, patients with advanced solid tumors were selected based on 1% PD-L1 expression in tumor or stromal cells (114). While a phase I/II study in urothelial carcinoma showed that patients with 25% PD-L1 expression in tumor or immune cells had higher response rates to durvalumab (115). In another study, circulating exosomal PD-L1 was reported to serve as a predictive biomarker of pembrolizumab response in patients with melanoma (113). Elevated levels of PD-L1 before treatment were associated with T cell exhaustion and reduced therapeutic benefit, while an increase during treatment was associated with T cell reinvigoration and enhanced antitumor immunity. These findings support the use of PD-L1 levels, including exosomal PD-L1, to stratify patients likely to benefit from ICIs, and further highlight its potential as a non-invasive blood-based marker for monitoring and predicting therapeutic outcomes during anti-PD-1 therapy. Importantly, exosomal PD-L1 also complements existing diagnostic tools. Unlike tissue-based PD-L1 immunohistochemistry, which is limited by intratumoral heterogeneity and insufficient biopsy samples (116), circulating exosomal PD-L1 offers a repeatable, minimally invasive alternative that captures dynamic changes during therapy. This position as a valuable adjunct to conventional assays, particularly in patients where tissue availability or sampling frequency is a challenge.

These insights into exosomal PD-L1 not only reinforce its prognostic and predictive utility, but also exemplify the broader clinical relevance of liquid biopsy approaches, which offer a non-invasive means to dynamically monitor tumor evolution and therapeutic response. For example, in breast cancer, exosomal miR-1246 was reported to distinguish patients from healthy individuals using a gold nanoflare probe, which demonstrated high sensitivity and single-molecule specificity at relatively low cost compared to conventional qRT-PCR, while also offering faster turnaround times (7). ELISA assays, such as those used for protein markers like PD-L1 (106), are cost-effective but have limited multiplexing capacity. In contrast, nanosensor-based approaches for miRNAs provide higher sensitivity and adaptability, making them promising for clinical use where precision, scalability, and affordability are essential. In colorectal cancer, Lui et al. (117) showed that CRC-secreted exosomal miR-1246 is internalized by hepatic stellate cells (HSCs), leading to their activation through the INSIG1/SREBP2/cholesterol metabolism axis, which reprograms the tumor microenvironment and promotes liver metastasis. Importantly, these findings suggest that exosomal miR-1246 could serve as a non-invasive biomarker for predicting colorectal cancer liver metastases. Similarly, in lung cancer, Huang and Qu (118) demonstrated that serum exosomal miR-1246 was significantly upregulated in non-small cell lung cancer (NSCLC) patients, correlated with lymph node metastasis and TNM stage, and acted as an independent prognostic factor for poor survival. ROC analysis confirmed its strong diagnostic performance, while dynamic changes in its levels reflected treatment response and recurrence. Together, these findings underscore the versatility of exosomal miR-1246 as a diagnostic and prognostic biomarker across multiple solid tumors, including breast, colorectal, and lung cancers. Furthermore, exosomal miR-105, miR-21, and miR-222 have shown promise as predictive markers for neoadjuvant chemotherapy and in the diagnosis of breast cancer (8). Furthermore, high levels of the exosomal protein CD82 have been associated with metastasis, likely reflecting its redistribution from tissues to exosomes during tumor progression (119). miR-210-3p, miR-5100, and miR-193a-3p were identified as novel biomarkers of lung cancer progression (120). In ovarian cancer, exosomal miR-200b and miR-200c have been reported to be associated with poorer overall survival, with their expression levels showing a significant correlation with CA-125 (Cancer Antigen 125) levels (121). Although miRNAs have been the main focus, long exosomal RNAs such as lncRNAs offer greater potential for tracking somatic mutations and gene expression changes. Exosomal lncRNA PCAT-1, detected in urine, has been proposed as an independent prognostic biomarker to assess relapse-free survival in patients with nonmuscle-invasive bladder cancer (122), further underscoring the potential of exosomes as liquid biopsies in cancer prognosis. Beyond bladder cancer, similar strategies are being explored in other solid tumors: for instance, exosomal lncRNA HOTAIR has been linked to poor prognosis and metastasis in breast cancer (123), while exosomal lncRNA MALAT1 has been shown to promote chemoresistance and predict outcomes in ovarian cancer (124). These findings highlight the broader applicability of exosomal lncRNAs as minimally invasive biomarkers for early detection, treatment monitoring, and therapeutic stratification across multiple cancer types.

While most ongoing exosome-based clinical trials focus on their diagnostic and prognostic potential, cancer-derived exosomes are increasingly recognized for their immunomodulatory roles, influencing antitumor immunity and opening new avenues for cancer therapy. Exosomes have garnered interest as therapeutic delivery vehicles due to their endogenous origin, low immunogenicity, ability to cross the blood–brain barrier, high target specificity and excellent biocompatibility. Nanosomes, an exosome–gold nanoparticle delivery system, were developed to deliver doxorubicin for lung cancer therapy (125). The study demonstrated an efficient intracellular distribution of doxorubicin and enhanced therapeutic efficacy in H1299 and A549 nonsmall cell lung cancer cells, highlighting the potential of exosome-engineered platforms for targeted cancer treatment. Similarly, glioblastoma and brain endothelial cell exosomes were loaded with paclitaxel and doxorubicin to facilitate transport across the blood-brain barrier to brain tumors in a zebrafish model (18). In another study, engineered exosomes (iExoSTINGa) were used to deliver the cyclic GMP-AMP small molecule STING agonist, resulting in enhanced antitumor immunity and suppression of subcutaneous tumor growth of B16F10 (19). Additionally, exosomes isolated from peripheral blood were successfully loaded with MAPK1 siRNA and used to deliver the siRNA into monocytes and lymphocytes, leading to targeted gene silencing (126).

Together, these studies underscore the versatility of cancer-derived exosomes as diagnostic tools and therapeutic platforms, further confirming their emerging role in precision oncology and immune modulation.

## Engineering and isolation of exosomes for cancer therapy

Building on their natural capacity for intercellular communication, IDEs are now being engineered using a variety of physical, chemical, and biological techniques to enhance their specificity, cargo capacity, and therapeutic efficacy.

### Exosome engineering strategies

These engineering strategies are critical to translating exosomes into clinically viable platforms. Various methods such as electroporation, sonication, transfection, and surface conjugation are used to load exosomes with therapeutic molecules, including nucleic acids, proteins, and drugs (17). **Table 2** summarizes the most commonly used methods, their mechanisms, and representative examples from the current literature.

**Table 2:** Exosome engineering and loading methods in cancer immunotherapy and targeted therapy.

Engineering methods	Cargo type	Cell type	Mechanism	Cancer type	Reference
Electroporation and vortexing	Galectin-9 siRNA, DOGEM (prodrug of gemcitabine), Indocyanine Green (ICG)	Bone marrow-derived mesenchymal stem cells (BM-MSCs)	pH-responsive release; synergistic chemotherapy, immunotherapy (T-cell activation), and phototherapy; galectin-9 silencing	Pancreatic cancer	48
Sonication + chemical modification	siRNA (KRASG12D)	Macrophages	Gene silencing of oncogenic KRAS to inhibit tumor growth	Pancreatic cancer	127
Exogenous incubation	STING agonist (cGAMP)	T cells	Activation of STING pathway to stimulate innate and adaptive antitumor immunity	Melanoma	19
Electroporation & folate decoration	Survivin siRNA	HEK293T-derived exosomes	Tumor targeting via folate, surviving knockdown, apoptosis	Cervical cancer	128
Incubation (drug diffusion)	Paclitaxel (PTX), Doxorubicin	Macrophages	Trans-BBB drug delivery to kill brain tumor cells	Glioblastoma	18

The compiled studies demonstrate the versatility of engineered exosomes as targeted delivery vehicles in cancer therapy, utilizing various types of cargo such as siRNAs, chemotherapeutic prodrugs, and immune agonists. Various engineering methods, such as electroporation, sonication, and incubation enable efficient loading and targeting of exosomes derived from mesenchymal stem cells, macrophages, and other cell types. These approaches collectively enhance therapeutic efficacy through mechanisms including gene silencing, immune activation, pH-responsive drug release, and improved tumor targeting, showing promise across multiple cancer types including pancreatic, melanoma, cervical, bladder, and glioblastoma.

### Exosome isolation methods

Exosome isolation is a critical step that ensures purity and functional integrity before downstream applications. Commonly used methods include ultracentrifugation, size-exclusion chromatography, and immunoaffinity capture, each with distinct advantages and limitations. **Table 3** provides an overview of these isolation strategies, emphasizing their mechanisms and the applications they have been investigated in.

**Table 3:** Exosome isolation methods in cancer immunotherapy and targeted therapy.

Isolation methods	Cargo type	Cell type	Mechanism	Application	Reference
Differential Ultracentrifugation	Untreated exosomes (native cargo)	Plasma, urine, cell culture supernatant	Sequential centrifugation at increasing speeds to remove cells, debris, and larger vesicles; final pelleting of exosomes at high speed (100,000×g)	Widely used standard method; biomarker studies; therapeutic applications	129, 130
Density- gradient ultracentrifugation	Untreated exosomes (native cargo)	Plasma, serum, cell culture supernatant	Separation of vesicles based on buoyant density using sucrose or iodixanol gradients; improved purity compared to differential UC	Functional and proteomic studies; cancer biomarker discovery	130, 131
Size-Exclusion Chromatography (SEC)	Untreated exosomes (native cargo)	Plasma, cell culture supernatant	Separation based on vesicle size through porous matrix; preserves vesicle integrity and function	Functional studies, therapeutic applications, biomarker analysis	132
Immunoaffinity Capture (IAC)	CD16 marker	Plasma	Antibody binding to highly enriched exosome surface proteins enables selective isolation	Linking exosome origin to immunoregulatory function; biomarker discovery; HNSCC, other cancers	133

Ultracentrifugation is known as the gold standard when it comes to exosomes isolation strategies (134). Differential ultracentrifugation and density-gradient approaches (including isopycnic and moving-zone methods) are the primary ultracentrifugation techniques traditionally employed for exosome isolation. Differential ultracentrifugation also known as simple ultracentrifugation or the pelleting method is the most widely used approach for exosome isolation, accounting for nearly half of reported studies (45.7%) (129). Its principle is straightforward: by applying increasing centrifugal forces, extracellular components in a fluid sample are sequentially separated according to their size, density, and shape. This method is favored for its ease of use, minimal technical expertise requirements, and suitability for processing large sample volumes without the need for complex pre-treatment (135). Despite this, extracellular fluids exhibit significant heterogeneity, and differential ultracentrifugation frequently results in the co-precipitation of microvesicles with non-vesicular entities like protein aggregates and lipoproteins (136). Consequently, this can result in low purity, potentially affecting subsequent applications (137). For example, Paolini and colleagues showed that exosomes isolated by this method exhibited poor and inconsistent biological activity compared to more purified samples (138). To improve exosome isolation, researchers have developed new centrifugation methods, among which density-gradient centrifugation is widely used to separate particles by density (131).

Isopycnic density-gradient centrifugation entails setting up a tube with layers of a biocompatible medium with varying densities, such as iodixanol or sucrose, arranged from highest density at the bottom to lowest at the top (139). The sample is carefully placed atop this gradient and subjected to extended ultracentrifugation (e.g., 100,000 × g for 16 hours). During this process, extracellular components like exosomes, apoptotic bodies, and protein aggregates move through the gradient until reaching their isopycnic position, where their buoyant density matches that of the medium surrounding them. Although density-gradient centrifugation is widely regarded as the most effective approach for obtaining highly pure exosomes for downstream applications, it cannot distinguish extracellular vesicles of similar buoyant density but different sizes from exosomes (e.g., microvesicles) (140). To address the challenges of isopycnic centrifugation, moving-zone (rate-zonal) density-gradient centrifugation enables separation of particles by both size and density (141). This method allows for the isolation of vesicles with similar densities but varying sizes, such as exosomes, large microvesicles, and viruses. In this technique, the gradient medium is less dense than any component in the sample, and the centrifugation duration must be meticulously managed to avoid all particles sedimenting at the bottom. To reduce exosome loss, a dense cushion is frequently placed at the tube’s base to maintain vesicles within the gradient while allowing denser particles to sediment.

Size-exclusion chromatography (SEC) is a size-based separation technique that isolates extracellular vesicles, including exosomes, by passing a biological sample through a column packed with a porous matrix (142). Larger vesicles are excluded from the pores and elute first, while smaller particles enter the pores and elute later, allowing gentle separation with minimal impact on vesicle structure and function. Within just a decade, several commercial SEC kits specifically designed for exosome isolation have been developed, including qEV (iZON) and PURE-EVs (Hansa Biomed). iZON has developed an automated exosome isolation system (qEV Automatic Fraction Collector) built on the SEC platform, incorporating weight-dependent fractionation and sample collection (143). This system enables fast, precise, and scalable exosome isolation, while reducing hands-on time and variability. SEC preserves the natural structure and biological activity of exosomes through passive gravity flow, avoiding the high shear forces and structural damage associated with ultracentrifugation (144). It enables rapid, simple, and reproducible isolation from small sample volumes without extensive pre-treatment, while physiological buffers maintain vesicle integrity. Compared to ultracentrifugation, SEC allows selection of defined vesicle subpopulations, minimizes sample loss, and achieves high yield, making it particularly suitable for functional and therapeutic studies (145).

Immunoaffinity capture leverages the specific binding between antibodies and proteins or receptors that are highly enriched on the surface of exosomes, allowing selective isolation from complex biological fluids (146). Common exosome markers include transmembrane proteins such as CD9, CD63, CD81, CD82, Rab5, Alix, and annexins, as well as other components like lysosome-associated membrane protein-2B, heat shock proteins, platelet-derived growth factor receptors, and lipid-related proteins (147–152). This approach underlies several commercial exosome isolation products, including the Exosome Isolation and Analysis Kit (Abcam), Exosome-Human CD63 Isolation Reagent (Thermo Fisher), and Exosome Isolation Kit CD81/CD63 (Miltenyi Biotec), providing high specificity while preserving vesicle functionality. Notably, immunoaffinity capture of CD3(+) (T cell-derived) and CD3(−) (tumor-derived) plasma exosomes from HNSCC patients showed that tumor-derived exosomes induce stronger T cell suppression, demonstrating the method’s ability to link exosome origin to immunoregulatory function and disease progression (153).

## Challenges and future directions

Despite promising preclinical results, several challenges hinder the clinical translation of exosome-based therapies. Standardization of exosome isolation and loading methods remains difficult, leading to variability in yield and cargo encapsulation efficiency (16). For instance, even though gradient ultracentrifugation can purify exosomes with minimal contamination, its processing volume is limited, requires expensive equipment, and demands highly trained personnel (154). Additionally, prolonged exposure to ultracentrifugal force can damage exosome structure and function, compromising downstream applications such as functional studies and drug development (155). Additionally, SEC’s key challenge is that exosome preparations often display a broader size distribution, particularly at the lower end, indicating contamination with similarly sized particles such as protein aggregates and lipoproteins (156). To address this, combined strategies such as SEC with ultrafiltration or ultracentrifugation have been employed, resulting in higher-purity exosomes while preserving their functional integrity. Furthermore, the immunoaffinity capture approach is highly specific and preserves exosome function, but it is limited to exosomes that express the target antigen on a large proportion of vesicles (146), and it can be costly, difficult to scale, and may miss subpopulations lacking the selected marker.

Challenges in exosome engineering include low cargo loading efficiency, instability or premature leakage of cargo, population heterogeneity, altered biological function, limited scalability and reproducibility, potential safety and immunogenicity concerns, and regulatory or manufacturing barriers that hinder clinical translation (157). In response to restricted loading capacity, active cargo loading techniques have emerged, but these can lead to exosome aggregation, membrane damage, and necessitate rigorous purification (158). Endogenous loading consists of directly inserting therapeutic cargo into exosomes via the donor cell. This can be accomplished by either incubating the parent cells with the cargo or using gene editing to enhance the expression of target molecules for later encapsulation (159).

In addition to optimizing cargo loading, challenges such as target specificity and off-target effects must be addressed to prevent unintended immune responses or toxicity. One potential strategy is using autologous tumor cells as the source of exosome production, which can reduce neutralization by the patient’s immune system and enhance therapeutic efficacy (17). While allogeneic engineered IDEs carry a higher risk of immunogenicity, autologous IDEs are generally better tolerated; however, both may still cause off-target effects on healthy cells, highlighting the need for precise targeting, rigorous safety evaluation, and careful design of therapeutic cargo (160).

Phase I clinical trials have initiated investigations into the potential of utilizing exosome-based therapies for cancer treatment. One specific study (NCT01550523) assessed glioma cell–derived exosomes that were engineered to carry an antisense molecule against the insulin-like growth factor I receptor (IGF1R), demonstrating both the feasibility and safety of exosome-based therapeutic delivery (161). In a separate trial (NCT01159288), exosomes derived from autologous dendritic cells (DEX) were used as a therapeutic vaccine for patients with metastatic melanoma, indicating safety and tolerability, yet lacking strong responses from CD4^+^ or CD8^+^ T cells. This underscores the necessity for further exploration into how exosome-mediated antigen presentation can be optimized (162).

Current and completed early-phase studies investigate a variety of therapeutic approaches, such as mesenchymal stromal cell-derived exosomes loaded with KRAS^G12D siRNA for treating metastatic pancreatic cancer (NCT03608631; 163), plant-derived exosomes used to transport curcumin for colon cancer therapy (NCT01294072; 164), exosomes sourced from autologous ascites combined with GM-CSF for colorectal cancer (165), and dendritic cell-derived exosomes evaluated as a maintenance immunotherapy following initial chemotherapy in non-small-cell lung cancer (166). Collectively, these studies highlight the diverse array of exosome-based strategies presently under clinical evaluation (**Table 4**).

**Table 4:** Current and finalized clinical trials concerning exosome-derived treatments in oncology.

NCT ID	Phase	Approach/cargo	Indication	Status/highlights	Reference
NCT01550523	I	Glioma exosomes carrying IGF1R antisense	Glioma	Feasibility and safety demonstrated	161
NCT01159288	I-II	Autologous DC-exosomes (DEX) vaccine	Metastatic melanoma/NSCLC	Tolerable; modest T cell activation	162
NCT03608631	I	MSC-derived exosomes with KRAS^G12D siRNA	Metastatic pancreatic cancer	Evaluating dose and safety	163
NCT01294072	I	Plant-derived exosomes delivering curcumin	Colon cancer	Safety/tolerability under investigation	164
—	I	Autologous ascites exosomes + GM-CSF	Colorectal cancer	Completed Phase I; showed antigen delivery	165
NCT01159288 (Phase II extension)	II	MHC class I & II–restricted antigens	NSCLC	Modest clinical benefit; median OS 15 mo; primary endpoint not met	166

Building on these early-phase studies, additional trials have demonstrated the feasibility and initial safety of engineered exosomes for targeted cancer therapy. However, translating these promising results into widespread clinical use is limited by challenges in large-scale manufacturing and quality control of clinical-grade exosomes (17). Standardized, GMP-compliant protocols are lacking, and scaling up while maintaining exosome purity, functionality, and batch-to-batch consistency remains a major bottleneck. Strategies such as automated bioreactor systems, advanced purification technologies, and the development of synthetic exosome mimetics are being explored to improve scalability, reproducibility, and safety for therapeutic applications (167).

Preliminary outcomes indicate that engineered exosomes are generally well-tolerated and capable of delivering therapeutic cargo, but immune responses and clinical efficacy have been variable. These findings underscore the need for optimized dosing strategies, improved targeting, and enhanced exosome engineering in future trial design to maximize therapeutic benefit, while advanced 3D ex vivo models and rigorous *in vivo* studies remain essential to fully evaluate pharmacokinetics, biodistribution, long-term safety, and therapeutic efficacy.

The future of exosome-based immunotherapy lies at the intersection of mechanistic insight, bioengineering, and clinical translation. While CDEs promote tumor progression by suppressing immune surveillance, this same pathway can be harnessed by engineering immune cell–derived exosomes to deliver tumor antigens, siRNAs, or checkpoint inhibitors that stimulate antitumor immunity. Combination strategies, such as pairing dendritic cell–derived exosomes with cytotoxic T cell activation, may offer more durable and systemic effects, though multiplexed immunotherapies must also address the complexity of the TME and ensure affordability at scale.

To support translation, advanced 3D ex vivo models (e.g., tumor–immune organoids) will be critical for testing efficacy, biodistribution, and safety under physiologically relevant conditions. Equally important is the standardization of isolation and engineering workflows, improving loading efficiency, cargo stability, and reproducibility for clinical-grade production. Emerging technologies, including AI, machine learning, and multi-omics, could accelerate this process by identifying predictive biomarkers, optimizing therapeutic payloads, and enabling personalized exosome therapies.

In conclusion, clinical success will depend on overcoming barriers in engineering, large-scale manufacturing, and regulatory standardization, while leveraging new models and computational tools to shift the TME balance toward immune activation and cancer control.
